# BCI-activated electrical stimulation in children with perinatal stroke and hemiparesis: A pilot study

**DOI:** 10.3389/fnhum.2023.1006242

**Published:** 2023-03-17

**Authors:** Zeanna Jadavji, Adam Kirton, Megan J. Metzler, Ephrem Zewdie

**Affiliations:** ^1^Cumming School of Medicine, University of Calgary, Calgary, AB, Canada; ^2^Department of Pediatrics, University of Calgary, Calgary, AB, Canada; ^3^Alberta Children’s Hospital Research Institute, Calgary, AB, Canada; ^4^Department of Pediatrics, Alberta Children’s Hospital, Calgary, AB, Canada; ^5^Department of Clinical Neurosciences, Alberta Children’s Hospital, Calgary, AB, Canada

**Keywords:** perinatal stroke, cerebral palsy, electrical stimulation (ES), brain computer interface, rehabilitation

## Abstract

**Background:**

Perinatal stroke (PS) causes most hemiparetic cerebral palsy (CP) and results in lifelong disability. Children with severe hemiparesis have limited rehabilitation options. Brain computer interface- activated functional electrical stimulation (BCI-FES) of target muscles may enhance upper extremity function in hemiparetic adults. We conducted a pilot clinical trial to assess the safety and feasibility of BCI-FES in children with hemiparetic CP.

**Methods:**

Thirteen participants (mean age = 12.2 years, 31% female) were recruited from a population-based cohort. Inclusion criteria were: (1) MRI-confirmed PS, (2) disabling hemiparetic CP, (3) age 6–18 years, (4) informed consent/assent. Those with neurological comorbidities or unstable epilepsy were excluded. Participants attended two BCI sessions: training and rehabilitation. They wore an EEG-BCI headset and two forearm extensor stimulation electrodes. Participants’ imagination of wrist extension was classified on EEG, after which muscle stimulation and visual feedback were provided when the correct visualization was detected.

**Results:**

No serious adverse events or dropouts occurred. The most common complaints were mild headache, headset discomfort and muscle fatigue. Children ranked the experience as comparable to a long car ride and none reported as unpleasant. Sessions lasted a mean of 87 min with 33 min of stimulation delivered. Mean classification accuracies were (*M* = 78.78%, SD = 9.97) for training and (*M* = 73.48, SD = 12.41) for rehabilitation. Mean Cohen’s Kappa across rehabilitation trials was *M* = 0.43, SD = 0.29, range = 0.019–1.00, suggesting BCI competency.

**Conclusion:**

Brain computer interface-FES was well -tolerated and feasible in children with hemiparesis. This paves the way for clinical trials to optimize approaches and test efficacy.

## Introduction

Perinatal stroke is a focal vascular brain injury that causes lifelong disability for millions (Nelson, 2007; Dunbar and Kirton, 2019). As the leading cause of hemiplegic cerebral palsy, and with no prevention possible, current research is largely geared toward understanding and improving motor recovery. Severity of hemiparesis can vary significantly between individuals with some children having extremely limited use of their affected arm and hand. As a result, these children may encounter difficulties with activities of daily living such as grooming, bathing, and feeding, in addition to age-appropriate participation in recreational activities. Current options are regrettably limited but increasingly informed by an improved understanding of how brain development occurs following such unilateral injuries at the beginning of life. Eloquent preclinical and human brain mapping studies are defining the developmental plasticity that occurs following perinatal stroke (Kirton, 2013b; Hilderley et al., 2019; Craig et al., 2021; Kirton et al., 2021).

In the motor system, bilateral corticospinal tracts present in equal proportions at birth are normally withdrawn from the ipsilateral side in the first years of life (Eyre, 2007). However, early unilateral injury may impair contralateral spinal innervation, resulting in abnormal persistence of ipsilateral connections and motor control of the affected limbs by the non-lesioned hemisphere (Staudt, 2007; Kirton, 2013a; Kirton et al., 2015). Different stroke subtypes represent a human model of developmental plasticity after early brain injury (Kirton and DeVeber, 2013). How such models relate to available rehabilitation therapies is increasingly understood. Constraint-induced movement therapy (CIMT) and bimanual therapies can be effective for some but require high doses and effect sizes are modest (Novak et al., 2013). Models have also defined targets for non-invasive neuromodulation, namely the non-lesioned primary motor cortex, where controlled clinical trials suggest additional efficacy (Kirton et al., 2015; Hilderley et al., 2019). There are currently no well-defined models of neuroplasticity in children with perinatal stroke as it relates to reorganization of cortical motor imagery and motor planning. Lack of understanding of how the young brain reorganizes after early injury creates a unique challenge when attempting to use mental imagery and intention as part of a rehabilitation paradigm.

Functional electrical stimulation (FES) represents an emerging rehabilitation option that has not been well- studied in hemiparetic children. FES is a form of neuromuscular electrical stimulation (NMES) that combines patient movement attempts with stimulation of target muscles *via* low intensity electrical currents facilitating repetition of impaired functional movements. The patient’s voluntary effort is an essential component of FES where cortical activation of sensorimotor areas are associated with functional improvement (Eraifej et al., 2017; Musselman et al., 2020). FES in adults has demonstrated improved upper extremity function and neuroplastic changes for post-stroke hemiparesis including improvements in activities of daily living (ADL) and is recommended by current best stroke rehabilitation practice guidelines (Eraifej et al., 2017; Musselman et al., 2020). Small studies combining therapy with FES in children with hemiplegic CP have suggested improved hand function with associated changes in cortical neurophysiology (Wright and Granat, 2000; Xu et al., 2012; Mukhopadhyay et al., 2015). How such peripheral approaches can be integrated with therapies targeting central mechanisms has not been explored in pediatric populations.

Brain computer interfaces (BCIs) represent a novel means by which cortical activity might be harnessed to enhance rehabilitation of hemiparesis. BCIs sample user brain activity and detect thought-induced changes to allow control of external effector devices (Wolpaw et al., 2002). Implanted BCI technologies have allowed completely paralyzed patients to use their cortical activity to control effector devices (Hochberg et al., 2012; Sharma et al., 2016). Non-invasive BCIs are rapidly advancing with many using electroencephalography (EEG) to sample and convert neural activity into control commands. More specific to hemiparesis, it has been hypothesized that re-connecting residual or “offline” motor planning systems with persistent connections to the paretic limb might facilitate recovery. BCI might therefore provide such a closed loop system where patients are able to plan and visualize desired movements by connecting detected motor imagery or intent to receive real-time movement-induced sensory and visual feedback (Bockbrader et al., 2018). A recent meta-analysis of such BCI-based stroke rehabilitation in adults examined such studies pairing BCIs with robotic limbs, exoskeletons, hand orthotics, visual feedback, and FES (Cervera et al., 2018). Six of the nine studies suggested clinically significant gains in motor function scores, the most significant of which were observed with BCI-FES. A randomized, blinded, controlled study of 27 adults with stroke described lasting improvements in upper extremity motor function and ipsilesional EEG connectivity after 10 h of BCI-FES training (Biasiucci et al., 2018). Smaller open-label studies using readily available BCI-FES systems also suggested possible benefits (Irimia et al., 2017, 2018). We have shown that school-aged children can effectively control simple BCI (Zhang et al., 2019) including those with perinatal stroke (Jadavji et al., 2021). Therefore, we conducted a phase 1 clinical trial to determine the safety, tolerability, and feasibility of BCI-FES in children with hemiparesis due to perinatal stroke.

## Materials and methods

Participants were recruited from the Alberta Perinatal Stroke Project (APSP), a population-based cohort of over 500 children (Cole et al., 2017). Inclusion criteria were: (1) MRI confirmed perinatal stroke, (2) age 6–18 years, (3) disabling hemiparetic cerebral palsy (defined as reduced functioning of the affected arm to a degree that the individual has goals for functional improvement), and (4) informed consent and assent. Each participant had reduced motor function of their affected hand, limiting their ability to participate in desired tasks and/or activities. Children with neurological comorbidities unrelated to stroke, multiple strokes, severe developmental delay, or unstable epilepsy were excluded. This study was approved by the University of Calgary Research Ethics Board.

### BCI-FES system

This trial was completed using the g.tec recoveriX system (g.tec, Schiedlberg, Austria), a commercially available BCI suite combining EEG recording and NMES. EEG was recorded *via* a 16 recording channel, gel- based headset. Recording electrodes were placed using standard 10-10 EEG locations at FC5, FC1, FCz, FC2, FC6, C5, C3, C1, Cz, C2, C4, C6, CP5, CP1, CP2, CP6 with a ground electrode placed at Fpz and a reference electrode on the participant’s earlobe. The most appropriate cap was chosen from a range of pediatric and adult sized small, medium and large options. Data was recorded using the g.USBamp amplifier (g.tec, Graz, Austria). EEG was sampled at 256 Hz and bandpass filtered at 0.5 Hz to 20 Hz to capture the alpha, beta and mu frequency ranges that are needed to calculate event -related desynchronizations (ERDs) (Miao et al., 2021). Data collected from the g.USBamp was converted to a 24 bit digital signal and transmitted to the computer. The data was further classified using linear discriminant analysis (LDA) and common spatial patterns (CSP) based on motor imagery induced synchronization. Signal quality was verified through a color-coded montage and raw EEG was monitored using the recoveriX software (g.tec, Graz, Austria). Users are unable to make modifications to the pre-set classification methods employed by the recoveriX system. The existing classification code is commonly used for sensory-motor rhythm-based BCI paradigms.

**FIGURE 1:**
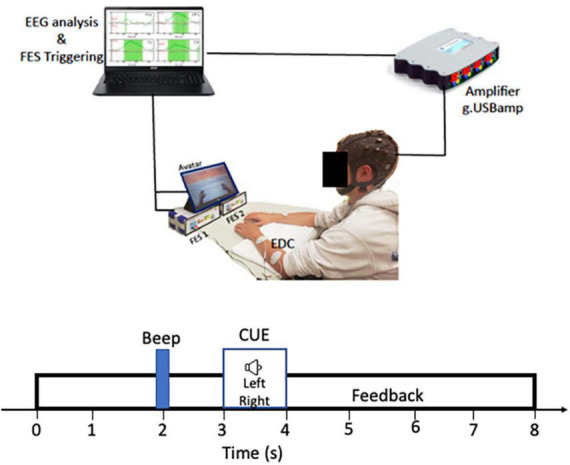
RecoveriX system set up: Participants were seated comfortably with arms positioned flat on a table directly in front of them. Functional electrical stimulation (FES 1 and FES 2) are g-estim units for left and right EDC electrical stimulation. The main computer controls both the FES triggering and EEG analysis.

Muscle stimulation was delivered bilaterally by two g.Estim electrical stimulators (g.tec, Graz, Austria), which generates rectangular bi-phasic, constant current pulses. A set of disposable “2 × 2” carbon rubber electrodes were applied to each arm to optimize contraction of the extensor digitorum communis (EDC) muscle and produce finger and wrist extension. The anodal electrode was placed on the proximal dorsal forearm near the elbow, and the cathodal electrode was placed on the distal dorsal forearm. Maximum output for current and voltage was ±60 mA and ±80 V with a frequency range of 1–100 Hz. The frequency of the NMES was slowly increased from 1 Hz to a maximum of 100 Hz. This frequency range was determined by the possible stimulator output range of the g.tec FES device to maintain safety and tolerability. A monitor depicting two virtual hand avatars was placed directly in front of the participant during testing for visual feedback which was delivered to the participants in synchrony with the electrical stimulation. The BCI-FES system set up is summarized in Figure 1.

### Testing

Participants completed two BCI sessions ranging from 1 to 2 h in length. Testing was completed in the Pediatric Brain Computer Interface Lab at the Alberta Children’s Hospital. Participants attended with a parent and were oriented to all equipment and procedures prior to beginning. At this time, participants were instructed on how to perform motor imagery by imagining wrist extension of their left and right hand. The set-up described above was established over 10–15 min. NMES parameters were individualized for each upper extremity.

NMES pulse width and frequency were then optimized to the level that was most comfortable while resulting in tetanic wrist extension. To measure perception threshold of electrical stimulation, children were asked to identify at what point they began to feel any sensation and this current amplitude value was recorded for each session (current perception threshold). To determine the amplitude to be used for FES, the current was increased until tetanic wrist extension of each hand was comfortably achieved and the current value was recorded. At all times, children were blinded to the electrical stimulation parameters. Given that the affected arm often has smaller muscle bulk and some degree of muscle atrophy we expected to see a difference in electrical stimulation parameters between both hands. Larger muscle bodies with more muscle fibers will require higher currents to produce muscle contraction (Enoka et al., 2020).

Each testing session consisted of 3 trials lasting approximately 15 min each. Each trial consisted of 80 runs, each run lasting 8 s. At the beginning of each run, participants heard a predefined sound, notifying them that a directional command was about to occur. After 2 more seconds, a “left” or “right” auditory command was given which signaled the participant to begin motor imagery, visualization of the extension of their left or right wrist and moving of their hand. At 1.5 s after the command was given, participants received visual feedback and electrical stimulation based on the assigned protocol: (1) Training or (2) Rehabilitation, which were always completed in the same order (see below). Length and timing of trials were pre-set by the recoveriX system and modifications were not possible. A classification accuracy, reported as a percentage, was generated by the recoveriX software upon completion of each trial. Classification accuracy is a measure of how accurately the BCI system can predict which motor imagery commands a participant is attempting. Participants were also asked whether they would like to end the session or continue onto the next trial and their reasons for wanting to stop were recorded.

### Training session

Participants completed the training protocol during session one during which they were required to successfully perform at least two of the three trials and achieve a classification accuracy above the significance level (*p* < 0.05), as calculated by the system. The two trials minimum is required to successfully produce a classifier. Using binomial distribution for a 5% significance level, the chance level was calculated to be 50% for the 2-class BCI (Müller-Putz et al., 2008). During training, participants received visual feedback and electrical stimulation during each run. Participants who were unable to successfully complete the training phase repeated the protocol in a second session until success was achieved. Once training was successfully completed, participants moved on to the rehabilitation session.

### Rehabilitation session

The classifier that was generated for each participant during the training protocol was used during the rehabilitation session. Participants were asked to complete as many of the three trials as possible. Set up and audio cues were delivered similarly as during training session. However, visual feedback and electrical stimulation were only delivered when the participants were able to generate distinguishable left and right motor imagery which matched the directional command as determined by the recoveriX system.

### Tolerability

A brief tolerability survey was administered between each trial to measure subjective, self-reported fatigue, level of enjoyment, and comfort during the task and session. Questions were scored on a Likert scale of 1–5. At the end of each session, participants completed a tolerability scale where they were asked to rank their enjoyment of the BCI experience against six other common childhood experiences.

### Attention and executive function questionnaires

Because BCI requires sustained, focused attention, we additionally collected parent ratings of attention and executive functioning to explore associations with BCI efficacy. Parents were asked to complete the attention-deficit hyperactivity disorder (ADHD) Rating Scale (ADHD-RS-IV) (Pappas, 2006) and the Behavior Rating Inventory of Executive Function (BRIEF) (Gioia et al., 2000). The ADHD-RS-IV is a well-validated, parent-rated questionnaire with high internal consistency and retest reliability and provides separate percentile scores for Hyperactivity-Impulsivity (ARS-H) and Inattention (ARS-I) (Pappas, 2006). Scores were converted to a percentile score using age- and gender -matched published norms, with higher percentile scores suggesting poorer performance (higher levels of parent-rated ADHD symptoms). The BRIEF is a standardized questionnaire with normative values based on 1,419 typically developing children. The global executive composite (GEC) is one of three summary indices and a valid and reliable measure of parent ratings of executive functioning. Higher scores indicate poorer parent ratings of executive functioning and a T-score ≥ 65 on any of the BRIEF measurements is considered clinically elevated.

### Hand function assessments

Two measures of hand function were included which was collected from a previous research study. The Box and Blocks Test (BBT) entails grasping a block from a box, transporting it over a barrier, releasing and then repeating that sequence as many times as possible in 60 s. The number of blocks was recorded for the affected and unaffected hands separately. The Assisting Hand Assessment (AHA) is a validated, play-based test measuring bimanual motor function for children with hemiplegic cerebral palsy. BBT scores and AHA l ogit scores were available for 11 of 13 participants. These two assessments were used as measures of upper extremity function where higher scores reflect better function (Jongbloed-Pereboom et al., 2013; Louwers et al., 2016). Hand function was not re-tested after rehabilitation as this study was designed to assess if our participants would be able to tolerate and operate a pre-existing BCI-NMES system. Participants were also only scheduled to complete a single rehabilitation session, which we would not expect to induce significant changes in motor functioning.

### Analysis

Independent and paired samples *t*-tests were used to compare mean and highest classification accuracies achieved during training versus during rehabilitation sessions. Pearson’s Correlations were performed to explore associations between (1) current perception threshold and session length, (2) muscle contraction current amplitude and session length, (3) age and classification accuracies, (4) Cohen’s Kappa and classification accuracies across Rehabilitation trials, (5) classification accuracies and Cohen’s Kappa scores vs AHA and BBT scores. Cohen’s Kappa was chosen as a measurement of BCI competency as it has been used to assess BCI ability in typically developing children (Cervera et al., 2018). Paired sample t-tests were performed to compare current perception threshold between affected and unaffected arm at the beginning and end of each session, as well as between the two sessions. The same test was performed to compare the current amplitude required for muscle contraction of the affected and unaffected arm. Statistical analysis and graph generation were performed SPSS 24 and SigmaPlot 12.5, respectively.

## Results

A total of 13 participants were recruited (69% male, mean [SD] 12.2[2.1] years, range 9–18 years). The study population is summarized in Table 1. Complete data from both sessions were collected from 11 participants who successfully completed the training protocol on day 1 and moved onto the rehabilitation session. One participant presented with abnormal EEG activity and the system was unable to generate a classifier. The participant did however attempt the training protocol on both days and all data aside from classification accuracy and Cohen’s Kappa were recorded and included in analyses. For another participant, we were only able to collect complete training data on day 2 due to technical failures with signal acquisition during their first session. This participant was unable to return for an additional session to complete data collection and was not included in the analysis. Our final sample size was therefore 11 participants with complete datasets and one additional participant with a partial dataset (omitted classification accuracy and kappa values).

**TABLE 1:** Participant demographics and reporting of adverse events.

Participant	Age (years)	Sex		Stroke type	Box and block test			Assisting hand assessment (logit)
					Affected hand	Unaffected hand		
1	10.5	M		Arterial	N/A	N/A		N/A
2	15.4	F		Venous	46	55		84
3	14.1	M		Venous	N/A	N/A		N/A
4	14.9	F		Venous	42	67		57
5	11.8	M		Venous	36	53		97
6	10.5	M		Venous	31	58		70
7	12.2	M		Arterial	28	53		58
8	12.2	M		Venous	41	68		81
9	10.1	M		Arterial	51	65		82
10	12.0	F		Arterial	18	54		46
11	9.9	F		Arterial	15	47		52
12	9.7	M		Venous	27	51		63
13	15.6	M		Arterial	13	59		32
	**Headache**	**Nausea**	**Head itching**	**Arm itching**	**Unpleasant tingling**	**Muscle fatigue**	**Muscle pain**	**Headset discomfort**
% of participants reporting during training	42%	0%	25%	8%	17%	50%	8%	58%
# mild/mod/sev	4/1/0	0/0/0	2/1/0	1/0/0	2/0/0	4/2/0	1/0/0	2/4/1
% of participants reporting during rehab	17%	0%	33%	0%	8%	33%	17%	33%
# mild/mod/sev	2/0/0	0/0/0	3/1/0	0/0/0	0/1/0	1/3/0	1/1/0	1/4/0

Percentage of children reporting each adverse event presented with the number of children ranking the event as mild, moderate or severe uncomfortable. The numbers included represent the number of participants that endorsed that particular adverse event. Participants were not counted more than once for a given adverse event.

### Time and trials

The average total time to complete sessions one and two, including set up, was 92 ± 19 and 82 ± 18 min. When considering protocol time alone, participants spent an average of 35 ± 8.5 and 31 ± 8.7 min attempting the training and rehabilitation trials. All participants completed the required minimum of two training trials and seven participants completed three trials. During rehabilitation, eight participants opted to end the study after completing two trials.

### Electrical stimulation parameters

For most participants, frequency (50 Hz) and pulse width (300 μs) were held constant for both arms during current perception threshold measurements and for trials performed after the most tolerable parameters were determined. Participant 1 had a high threshold (>5 mA higher than the mean current of 9.32 mA) for muscle contraction and their parameters were adjusted to a pulse width of 381 and 371 μs for more comfortable stimulation. No significant difference was identified in the current perception threshold between the affected (*M* = 3.44 mA, SD = 1.43, range = 2–7 mA) and unaffected (*M* = 3.23 mA, SD = 1.25, range = 2–9 mA); *t*(51) = 1.32, *p* = 0.20) hands. There was no difference in current perception threshold between the beginning and end of each session. Participants showed a higher current perception threshold of the affected hand for session 2 (*M* = 3.92 mA, SD = 1.49) as compared to session 1 (*M* = 3.08 mA, SD = 0.93); *t*(25) = −3.53, *p* = 0.002). Stimulation parameters for muscle contraction varied significantly between the affected (*M* = 8.10 mA, SD = 2.73, range = 4–14 mA) and unaffected (*M* = 9.32 mA, SD = 2.70, range = 3–13.8); *t*(61) = −7.08, *p* < 0.01) hands. No correlation was observed between current perception threshold (all *p* > 0.10) or current for muscle contraction (all *p* > 0.14) and duration of the BCI session.

### Classification accuracy and Cohen’s Kappa

**FIGURE 2:**
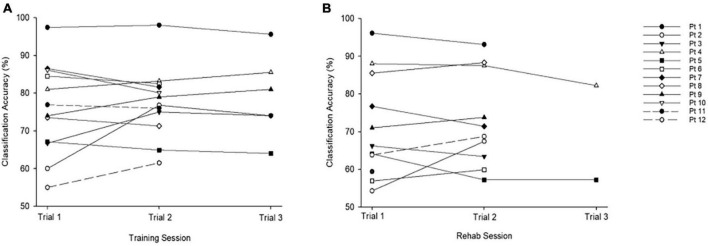
Classification accuracies across training and rehabilitation sessions. **(A)** Average accuracy across all training trials was *M* = 76.91%, SD = 10.78, range = 55–96%. **(B)** Average accuracy across all rehabilitation trials was *M* = 70.85%, SD = 12.21, range = 57–88%. The chance level is 50%.

Mean classification accuracies achieved for training and rehabilitation trials were comparable: training (*M* = 76.91% (SD = 10.78, range = 55–96%) and rehabilitation (*M* = 72.72%, SD = 12.72, range = 57–88%; *t*(10) = 1.19, *p* = 0.14). Highest classification accuracies for training (*M* = 78.78%, SD = 9.97) and rehabilitation (*M* = 73.48, SD = 12.41; *t*(10) = 1.6, *p* = 0.14) sessions were also comparable. No correlation was observed between age and classification accuracy for either the training or rehabilitation protocols. Of the 12 participants who generated classification accuracies during training, five improved their scores between their first and final trial (Figure 2). Of the 11 participants who generated classification accuracies during the rehabilitation session, five improved their scores between initial and final trials (Figure 2).

**FIGURE 3:**
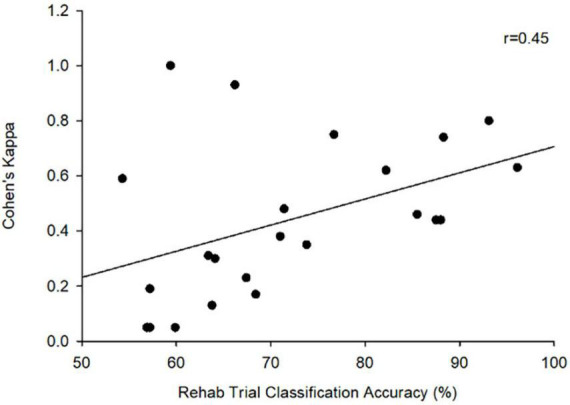
Cohen’s Kappa vs. rehabilitation classification accuracy (*r* = 0.45, *p* < 0.05).

Since participants could only trigger visual feedback and electrical stimulation based on their motor imagery during rehabilitation trials, Cohen’s Kappa was only calculated for these sessions. Across all trials, participants achieved an average Cohen’s Kappa of (*M* = 0.43, SD = 0.29). Seven participants were able to achieve a highest Kappa of at least 0.40, the threshold commonly employed in adult studies to suggest BCI competency (Scherer et al., 2013; Jeunet et al., 2016). There was a moderate correlation between Cohen’s Kappa scores and classification accuracy during the rehabilitation trials (*r* = 0.45, *p* = 0.032) (Figure 3).

### Attention, executive function, and hand function

**FIGURE 4:**
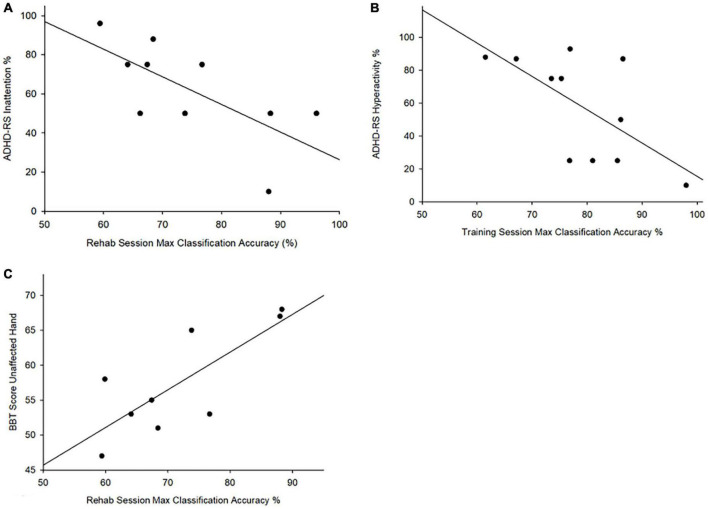
Attention and box and block test scores vs. maximum classification accuracy. correlation between **(A)** ADHD-RS Inattention% and maximum classification accuracy achieved rehabilitation (*r* = –0.690, *p* = 0.027); **(B)** ADHD-RS hyperactivity% and maxim classification accuracy achieved during training (*r* = –0.646, *p* = 0.032); **(C)** Correlation between BBT scores for the unaffected hand and the highest achieved classification accuracy during rehabilitation (*r* = 0.78, *p* = 0.01).

Attention-deficit hyperactivity disorder rating scale scores for Hyperactivity-Impulsivity (ARS-H) were negatively correlated with highest classification accuracies achieved during training (*r* = −0.646, *p* < 0.05) such that lower ARS-H scores (fewer ADHD hyperactivity-impulsivity symptoms) were associated with better classification accuracies. Inattention (ARS-I) scores were also negatively correlated with highest classification accuracy achieved during rehabilitation (*r* = −0.690, *p* = 0.05) (Figure 4). No correlations were observed between BRIEF T Scores and Cohen’s Kappa or highest classification accuracy achieved during each session. No correlations were observed between classification accuracies and the AHA or BBT affected hand scores. BBT scores for the unaffected hand were correlated with highest achieved classification accuracy during rehabilitation (*r* = 0.78, *p* = 0.01) (Figure 4).

### Safety and tolerability

**FIGURE 5:**
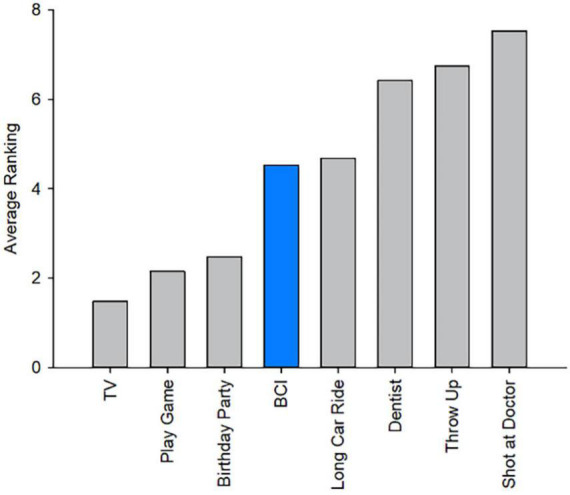
Brain computer interface ranking among other childhood experiences: Average ranking comparing the experience of BCI-FES to common childhood activities. Ranking listed from most to least enjoyable (1–8).

There were no serious adverse events. Headset discomfort was the most frequently reported complaint during BCI sessions (58%), followed by muscle fatigue (50%). Reported discomfort ranged from mild to moderate with only one instance of severe headset discomfort which was mitigated upon removal of the EEG cap (Table 1). On a scale of 1 (very easy) to 5 (very hard), the average rating of the level of mental and physical demand during training was (*M* = 2.06, SD = 0.81) and (*M* = 1.74, SD = 0.82). During rehabilitation, average rankings of mental and physical demand to complete the task were (*M* = 2.20, SD = 0.97) and (*M* = 1.76, SD = 0.86). Average ratings of fatigue, from 1 (not tired) to 5 (very tired), during training and rehabilitation were comparable at *M* = 2.48 (SD = 0.96) and *M* = 2.76 (SD = 1.01). Average ratings of how pleasant participants found using the BCI-FES device from 1 (unpleasant) to 5 (pleasant) across all trials was *M* = 3.88, SD = 0.94. Participants found the rehabilitation trials (*M* = 4.20, SD = 1.00) more pleasant than the training session (*M* = 3.60, SD = 0.71; *t*(24) = −3.0, *p* < 0.001). Of those who were unable to complete all three training trials, the most commonly reported reason for choosing to stop was that they felt too mentally tired to continue. Four children chose to end the session because the activity was not engaging enough, two reported that they did not like the feeling of the electrical stimulation, and two stopped due to headset discomfort. When participants were asked to rank their BCI experience among seven other common childhood experiences from most to least enjoyable, average rankings suggested BCI sessions were preferable to a long car ride but not as enjoyable as a birthday party (Figure 5).

## Discussion

Our pilot trial supports the potential feasibility of BCI coupled FES for rehabilitation of hemiparesis in children. We demonstrate preliminary evidence that school-aged participants with disabling hemiparesis due to perinatal stroke can operate a commercially available BCI-FES system with average classification accuracies of greater than 70% and BCI competency across both training and rehabilitation protocols. Potential challenges identified include user fatigue, neurophysiology, comfort and attention. In the face of limited rehabilitation options for this common clinical syndrome, additional trials may be indicated.

Functional electrical stimulation has an established ability to improve functional outcomes in hemiparetic adults with stroke (Eraifej et al., 2017). Current stroke rehabilitation best practice guidelines consider FES to have grade A level evidence in this regard (Hebert et al., 2016). Evidence to support integration of BCI to facilitate patient activation of electrical stimulation and augment neuroplasticity *via* closed looped feedback is much less- established but early evidence is encouraging (Mukaino et al., 2014).

Evidence for FES in children with hemiparetic CP is not as abundant but increasing. One study of children with CP undergoing 30 min of FES therapy 5 times a week for 6 weeks described significant improvements on assessments of upper limb function (Yıldızgören et al., 2014). While previous research has demonstrated that FES protocols can be tolerated in children with hemiplegic CP, some may require more time to become comfortable with the sensation (Garzon et al., 2018). Studies in CP often combine electrical stimulation with other forms of therapy, however duration of treatment ranges on the order of weeks to months (Merrill, 2009). The dosage of muscle stimulation used for adult stroke rehabilitation varies widely but higher daily dosage appears to be related to greater recovery of arm function (Hsu et al., 2012). Our results here suggest that participants are able to complete approximately 30–35 min of BCI-FES per session. This technology appears feasible and well- tolerated in children, however it may be difficult to maintain engagement over longer periods of time, including durations suggested to be required for effective FES therapy. These will be important considerations for future trials.

Our results supplement growing evidence that children can successfully operate BCI. Previous studies have identified that a Cohen’s Kappa of at least 0.40 suggests BCI competency. While there was a large range in individual Cohen’s Kappa, average scores across the Rehabilitation session in our population was 0.43. This suggests that, on average, our pediatric subjects with perinatal stroke achieved BCI performance levels deemed as competent (Scherer et al., 2013; Jeunet et al., 2016). The variation in performance is likely multifactorial and attributed to variables including but not limited to attention, stroke type and individual neuro-reorganization. Future studies with larger samples would benefit from assessing these variables in isolation. All 13 children were able to complete the training protocol however a classifier was only created for 12 due to one participant presenting with abnormal EEG activity. Despite this, we found that our participants can tolerate the minimum training duration required to generate a classifier using this system. These results are consistent with previous studies demonstrating that both typically developing school-aged children (Zhang et al., 2019) and those with perinatal stroke can successfully operate commercially available BCI systems that are simple to set up and use. 27 Participants achieved average classification accuracy of greater than 70% however, no improvement in individual accuracies were noted across trials. Classification accuracy is a function of both the supervised machine learning of the classification algorithm as well as the improvement in user learning. Our pilot study did not include enough repetitions to induce learning improvements but future studies will aim to have participants complete at least 2 weeks of training.

There remains however a paucity of BCI studies focused in pediatric populations, particularly those with neurological disabilities (Letourneau et al., 2020). Increased awareness and engagement of pediatric participants will hopefully drive progress in practical applications such as neuromotor rehabilitation.

User fatigue was a significant concern. We have shown that fatigue is not only common in children with perinatal stroke but may also be associated with cortical neurophysiology (Wrightson et al., 2020). Specifically, children with ipsilateral corticospinal tract projections originating from the contralesional hemisphere demonstrated higher levels of fatigue. While our study did not account for these differences in neurophysiology between participants, future studies would benefit from exploring the effects of motor reorganization on fatigue in BCI rehabilitation. Future considerations may also be given to the impact of mirror movements on BCI ability. A subset of our participants were known to experience mirror movements though this was not systematically assessed in the context of our study. Potentially even more relevant for this current study, we recently demonstrated that fatigue in this population appears to be related to the functional connectivity of the sensorimotor prediction network (Wrightson et al., 2020). Average ratings of fatigue and perceived mental or physical demand during each session were relatively low and did not differ between sessions. However, participants most often indicated that they chose to stop because they felt tired or found the task non-engaging. While there has been minimal research on BCI fatigue, one study in adults suggested that prolonged motor imagery often results in significant mental fatigue (Talukdar et al., 2019). A possible explanation for participants completing fewer rehabilitation sessions may be because visual feedback and electrical stimulation was only provided when the correct motor imagery is detected. The rehabilitation session required more attention and engagement to elicit feedback and was therefore potentially more inducing of fatigue. Muscle fatigue was reported by half of the participants and an important consideration for future studies. It is possible that longer rest times between electrical stimulation may combat muscle fatigue however this would result in an increased total duration of the BCI session and the potential to further exacerbate mental fatigue. Consideration must also be given to whether the number of electrical stimulations received is effective for inducing improvements in motor function. On average, children achieved relatively high classification accuracies across both sessions but elevated measures of inattention and hyperactivity were correlated with poorer performance. It is expected that maintaining focus, including during motor imagery, is likely to require sustained attention. Our findings are somewhat in contrast to previous studies where self-reported attention levels were not highly correlated with BCI classification accuracy in healthy adults (Myrden and Chau, 2015). Other preliminary studies have also suggested that BCI training may actually help improve attention function in children with ADHD (Qian et al., 2018; Lim et al., 2019). Data, not yet published, from a previous study by our group suggests that rates of attentional disorders are more common in children with perinatal stroke, suggesting this issue may require additional consideration in this specific population. How this might manifest in each individual would also be expected to differ. Participant 6 had exceptionally low Cohen’s Kappa scores. While they achieved classification accuracies of 82–85% during training, these dropped drastically to 56–60% in the rehabilitation session. This participant was extremely distracted during the second session, chose to stop after two trials, and expressed that he found the activity uninteresting. Maintaining participant engagement in a repetitive task with limited feedback is challenging, particularly in children. Future BCI-FES protocols might consider gamification of these systems to help sustain user attention and motivation, a concept supported by data from our clinical BCI program for children with severe neurological disability (Jadavji et al., 2022).

Disease specificity is a strength of our study. Many rehabilitation trials include only children with the unspecified syndrome of cerebral palsy or perhaps unilateral/hemiparetic CP but even these may constitute a wide variety of brain lesions. In contrast, perinatal strokes are focal, unilateral brain injuries acquired at specific times in otherwise healthy brains, constituting an ideal human model of developmental plasticity (Kirton et al., 2021). In keeping with this specificity, it is important to consider the potential effects of brain reorganization after early brain injury that may have occurred across subjects. Motor imagery itself may be difficult in such children given that they have always had absent or very impaired movement of their affected limb throughout their life. This may also explain our findings of correlation between scores on motor functioning of the unaffected hand and classification accuracy. It is possible that children who demonstrate better baseline motor functioning are also better able to visualize movements required for motor imagery. Most children with perinatal stroke manifest major differences in the developmental organization of their motor system, often engaging major components of the motor system in the non-lesioned hemisphere (Alawieh et al., 2017; Craig et al., 2018, 2019). How this disrupted developmental plasticity impacts motor imagery systems is poorly understood though abnormalities in more generic CP populations are described (Errante et al., 2019). Source localization analysis would be one approach that might enhance understanding of mechanisms underlying motor imagery in individual children. This may help to inform more personalized BCI systems in the future. We used a generic electrode montage in this study, unable to take into consideration the characteristics of what are often large cortical lesions in this population. As this was the first research study to explore BCI-NMES in a pediatric stroke population we avoided modifications to the software and system used. We did not alter the number or placement of EEG electrodes to avoid introducing confounding variables that were not the focus of this initial tolerability and feasibility study. Personalizing electrode montage to be sure cortical tissue is approximated to the sensor, or even further using advanced imaging or other means to localize motor intent signals more specifically, might facilitate BCI performance at the level of the individual. Adjustments to electrode placement may also mitigate headset discomfort to some extent. Future studies may consider alternate EEG cap designs to promote a more comfortable participant experience.

Our results support the potential use of BCI-FES in children with hemiplegic CP resulting from perinatal stroke while identifying important considerations for future trials.

## Data availability statement

The raw data supporting the conclusions of this article will be made available by the authors, without undue reservation.

## Ethics statement

The studies involving human participants were reviewed and approved by the Conjoint Health Research Ethics Board of the University of Calgary (REB15-2567). Written informed consent to participate in this study was provided by the participants’ legal guardian/next of kin.

## Author contributions

ZJ conceptualized and designed the study, collected the data, conducted the analyses, and drafted and revised the initial manuscript. AK, MJM, and EZ conceptualized and designed the study and reviewed and revised the manuscript. EZ provided the primary technical support and participated in the data collection. All authors contributed to the article and approved the submitted version.
